# Boron neutron capture therapy: moving towards targeted therapy for locally recurrent head and neck squamous cell carcinoma

**DOI:** 10.1186/s40779-019-0224-7

**Published:** 2019-10-25

**Authors:** Ying Sun

**Affiliations:** Department of Radiation Oncology, Sun Yat-sen University Cancer Center, State Key Laboratory of Oncology in South China, Collaborative Innovation Center for Cancer Medicine, Guangdong Key Laboratory of Nasopharyngeal Carcinoma Diagnosis and Therapy, Guangzhou, 510060 China

**Keywords:** Locally recurrent head and neck squamous cell carcinoma, Boron neutron capture therapy, Treatment efficacy

## Abstract

Locally recurrent head and neck squamous cell carcinoma (HNSCC) is often unresectable, and a repeat course of radiotherapy is associated with incremental toxicities. Boron neutron capture therapy (BNCT) is a novel targeted radiotherapy modality that can achieve a high dose gradient between cancerous and adjacent normal tissues. However, the relationships among the dose resulting from BNCT, tumor response to BNCT, and survival are not completely understood. Recently, a study published in *Radiotherapy and Oncology* investigated the efficacy of BNCT in the treatment of patients with locally recurrent HNSCC and the factors associated with favorable treatment response and survival. In this article, the findings, strengths and limitations of this study are discussed in depth, and the significance of the study and motivations for future research are highlighted.

## Main text

Recurrent disease is an obstacle to long-term survival in patients with head and neck squamous cell carcinoma (HNSCC). Approximately 15%~ 50% of HNSCC patients underwent surgery and/or radiotherapy experience local relapse [1]. Locally recurrent HNSCC poses as a therapeutic challenge for multiple reasons: 1) it is often considered unresectable; 2) reirradiation is associated with incremental toxicity; and 3) most patients are not sensitive to chemo- and immuno-therapy [2]. Some radiotherapeutic strategies that allow the delivery of high retreatment radiation doses without affecting nontumorous tissues, such as intensity-modulated radiotherapy and stereotactic radiotherapy, may improve tumor control rates in locally recurrent HNSCC patients [3–6]. However, the prognosis of these patients is still very poor and new effective treatments are urgently needed [4, 7].

Boron neutron capture therapy (BNCT) is a novel targeted radiotherapy for tumor cells that preferentially accumulate drugs containing the nonradioactive boron-10 [8]. BNCT is based on the nuclear capture and fission reactions that occur when boron-10 is irradiated with neutrons to yield linear energy transfer alpha particles and recoiling lithium-7 nuclei [9]. The short range of this reaction limits the damage to only tumor cells without affecting normal cells, even if the two types of cells are mingled at the tumor margin. This property allows BNCT to treat cancers that are inoperable or locally recurrent [10], such as HNSCC [11], high-grade gliomas [12], and melanoma [13]. In the treatment of HNSCC, the tolerance of the adjacent critical tissues usually limits the maximum dose delivered by BNCT [14, 15]. Presently, the relationships among the BNCT radiation dose, tumor response to BNCT, and survival are still unknown. In a study recently published in Radiotherapy and Oncology, entitled “Boron neutron capture therapy for locally recurrent head and neck squamous cell carcinoma: An analysis of dose response and survival”, Koivunor and colleges [16] investigated the efficacy of BNCT in the treatment of patients with locally recurrent HNSCC and the treatment response and survival associated factors.

In this study, 79 patients with locally recurrent HNSCC, who were treated with BNCT in Finland, between February 2003 and January 2012 were included. The prior treatments of these patients included surgery and/or radiotherapy to a median cumulative dose (66 Gy) administered with or without chemotherapy. The authors evaluated the tumor response using the RECIST v.1.0 criteria. Forty (50.6%) patients received two times of BNCT treatment, and 39 (49.4%) were treated once. Between the two treatments, the median time interval was 6 weeks. The tumor responses of 69 patients were evaluable. Forty-seven (68.1%) of them had treatment response, including 25 (36.2%) with complete response and 22 (31.9%) with partial response. Seventeen (24.6%) patients had stable disease lasting for a median of 4.2 months. Five (7.2%) patients had disease progression. The patients received two times of BNCT treatment responded more often than those received only one time (78.4% vs. 56.3%; *P* = 0.049). The median time without local progression was 9 months, and 35% of the entire cohort were free from local progression 2 years after BNCT. Additionally, the median survival time and 2-year overall survival rate were 10 months and 21%, respectively.

Some exciting findings of this study are as follows: 1) most patients with local recurrent HNSCC responded to BNCT; 2) a high minimum dose delivered to the tumor was a key predictive factor for treatment response, and the number of BNCT treatments was a minimally important factor for progression-free survival and overall survival; 3) tumor size < 25 cm^3^ was found to be a favorable prognostic factor for survival and achieving complete response; and 4) the minimum dose to the gross tumor volume was associated with the survival rates.

This study was the first to examine the relationships between the tumor dose from BNCT and treatment outcomes in locally recurrent HNSCC patients. The response assessments and follow-up schedules were strictly performed according to the institutional guidelines. Therefore, the patient cohort can be considered representative and the data robust. However, this study is a retrospective study; thus, some key statistics on critical factors such as human papilloma virus infection, adverse effects related to BNCT, and treatment-related deaths were not measured or recorded, and the authors could not control the outcome assessment. This study provides important evidence-based grounds for initiating randomized clinical trials to compare the efficacy of BNCT to other radiotherapy modalities; these studies are urgently needed to determine the better therapies or alternatives to improve the survival of these patients.

## Data Availability

Not applicable.

## Abbreviations

BNCT: Boron neutron capture therapy
HNSCC: Head and neck squamous cell carcinoma

## References

[1] Bourhis J, Le Maitre A, Baujat B, Audry H, Pignon JP (2007). Individual patients’ data meta-analyses in head and neck cancer. Curr Opin Oncol.

[2] Chang JH, Wu CC, Yuan KS, Wu ATH, Wu SY (2017). Locoregionally recurrent head and neck squamous cell carcinoma: incidence, survival, prognostic factors, and treatment outcomes. Oncotarget.

[3] Rwigema JC, Heron DE, Ferris RL, Andrade RS, Gibson MK, Yang Y (2011). The impact of tumor volume and radiotherapy dose on outcome in previously irradiated recurrent squamous cell carcinoma of the head and neck treated with stereotactic body radiation therapy. Am J Clin Oncol.

[4] Vargo JA, Ward MC, Caudell JJ, Riaz N, Dunlap NE, Isrow D (2018). A multi-institutional comparison of SBRT and IMRT for definitive reirradiation of recurrent or second primary head and neck cancer. Int J Radiat Oncol Biol Phys.

[5] Ohnleiter T, Truntzer P, Antoni D, Guihard S, Elgard AM, Noel G (2017). Prognostic factors for head and neck cancer reirradiation: a systematic review. Cancer Radiother.

[6] Siddiqui F, Patel M, Khan M, McLean S, Dragovic J, Jin JY (2009). Stereotactic body radiation therapy for primary, recurrent, and metastatic tumors in the head-and-neck region. Int J Radiat Oncol Biol Phys.

[7] Ward MC, Riaz N, Caudell JJ, Dunlap NE, Isrow D, Zakem SJ (2018). Refining patient selection for reirradiation of head and neck squamous carcinoma in the IMRT era: a multi-institution cohort study by the MIRI collaborative. Int J Radiat Oncol Biol Phys.

[8] Barth RF, Mi P, Yang W (2018). Boron delivery agents for neutron capture therapy of cancer. Cancer Commun.

[9] Barth RF, Coderre JA, Vicente MG, Blue TE (2005). Boron neutron capture therapy of cancer: current status and future prospects. Clin Cancer Res.

[10] Barth RF, Zhang Z, Liu T (2018). A realistic appraisal of boron neutron capture therapy as a cancer treatment modality. Cancer Commun.

[11] Wang LW, Liu YH, Chou FI, Jiang SH (2018). Clinical trials for treating recurrent head and neck cancer with boron neutron capture therapy using the Tsing-Hua open Pool reactor. Cancer Commun.

[12] Miyatake SI, Kawabata S, Hiramatsu R, Kuroiwa T, Suzuki M, Ono K (2018). Boron neutron capture therapy of malignant Gliomas. Prog Neurol Surg.

[13] Hiratsuka J, Kamitani N, Tanaka R, Yoden E, Tokiya R, Suzuki M (2018). Boron neutron capture therapy for vulvar melanoma and genital extramammary Paget's disease with curative responses. Cancer Commun.

[14] Coderre JA, Morris GM (1999). The radiation biology of boron neutron capture therapy. Radiat Res.

[15] Gonzalez SJ, Santa Cruz GA (2012). The photon-isoeffective dose in boron neutron capture therapy. Radiat Res.

[16] Koivunoro H, Kankaanranta L, Seppala T, Haapaniemi A, Makitie A, Joensuu H (2019). Boron neutron capture therapy for locally recurrent head and neck squamous cell carcinoma: an analysis of dose response and survival. Radiother Oncol.

